# Medico-Economic and Medico-Social Evaluation of Two Types of Treatment for Clubfoot in France: A Prospective Study Using a Mixed Methods Approach

**DOI:** 10.3390/healthcare14203519

**Published:** 2026-10-19

**Authors:** Anaïs Le Jeannic, Belkacem Zenati, Clément Picot-Ngo, Sophie Guilmin-Crépon, Maria Texeira, Agathe Turpin, Priscilla Boizeau, Arnaud Nze Ossima, Corinne Alberti, Isabelle Durand-Zaleski, Ana Presedo, on behalf of the MESPIEDS Investigators

**Affiliations:** 1 Unité de Recherche Clinique en Économie de la Santé (URC Eco), Assistance Publique-Hôpitaux de Paris, 1 Place du Parvis Notre-Dame, F-75004 Paris, France; anais.le-jeannic@aphp.fr (A.L.J.); isabelle.durand-zaleski@aphp.fr (I.D.-Z.); 2 Inserm, ECEVE, Université Paris Cité, F-75010 Paris, France; clement.picot-ngo@inserm.fr (C.P.-N.);; 3 Unité de Recherche Clinique, AP-HP, Hôpital Universitaire Robert Debré, F-75019 Paris, France; sophie.guilmin-crepon@aphp.fr (S.G.-C.);; 4 Unité REPERES, AP-HP, Hôpital Universitaire Robert Debré, F-75019 Paris, France; 5 AP-HP, Unité D’épidémiologie Clinique, Inserm, CIC-EC 1426, Université Paris Cité, F-75010 Paris, France; 6 INSERM 1153 Centre de Recherche en Epidémiologie et Statistiques (CRESS), Sorbonne Paris Cité, F-75004 Paris, France; 7 Service de Chirurgie Pédiatrique Orthopédique, AP-HP, Hôpital Universitaire Robert Debré, F-75019 Paris, France

**Keywords:** cost-consequence, clubfoot, Ponseti method, French functional method, interview

## Abstract

**Background/Objectives**: Congenital clubfoot is the most frequent malformation in pediatric orthopedics. In France, two treatments can be proposed: the Ponseti method and the functional method. The objective was to conduct a medico-economic and -social evaluation of those methods. **Methods**: Medico-economic: a cost-consequences analysis was undertaken from the societal perspective, based on the Dimeglio score at 24 months in four pediatric hospitals. Medico-social: interviews were conducted with parents (*n* = 16) and healthcare professionals (*n* = 16). **Results:** One hundred patients were included between January 2014 and December 2016. Whatever the treatment method, over 90% of patients presented with benign clubfoot (i.e., Dimeglio score < 5/20) at 24 months, based on multiply imputed estimates. The Functional method was associated with higher direct medical and non-medical costs (€3340–4061 vs. 889–2318; €6275–5968 vs. 576–726), and indirect costs (€15,441–4672 vs. 1416–1786), contrary to out-of-pocket expenses (€0–1 vs. €322–546). Family testimonies underscored the importance of communication from healthcare providers to reassure and guide parents. Although the choice is influenced by professionals, trust and active engagement of families are essential for adherence to treatment and its long-term success. The Ponseti method combined effectiveness with a lower perceived burden for families. **Conclusions**: The Ponseti method was the costliest for parents, yet more manageable. However, the most important factor is to choose a method adapted to the family in order to achieve the best degree of compliance.

## 1. Introduction

### 1.1. Background

Idiopathic clubfoot is one of the most frequent reasons for consultation in pediatric orthopedics. This congenital malformation, of undetermined etiology but with a genetic component, has a prevalence in Europe ranging from 1 to 2 per 1000 births [1]. Boys are more often affected than girls (3/1) [2], and the presentation is bilateral in 50% of cases. Diagnosis is commonly done during the second ultrasound around 20 weeks of pregnancy, enabling treatment to be planned before birth. With appropriate treatment, started in the first weeks of life, a complete cure is possible with no functional repercussions in adult life.

Several treatment modalities have been proposed over time. Surgical correction as the primary method of treatment has been abandoned due to the poor results demonstrated after long-term follow-up studies [3,4,5]. Indeed, despite satisfactory anatomical correction, pain and joint stiffness were often present in adulthood, with repercussions on sports and daily life activities. In the nineties, Dr. Ignacio Ponseti took up and perfected the method of treatment with successive casts (PM), describing a very precise methodology. Early success is obtained in over 90% of patients with a risk of relapse estimated to vary between 10% and 40% across reported series [6]. By virtue of the good results published, this type of treatment rapidly gained a worldwide following [6,7]. The feasibility, reproducibility and low cost of plaster cast treatment made it possible to spread clubfoot treatment to countries with less favorable socio-economic characteristics [8].

However, the emotional burden on families, particularly around the time of diagnosis, and the role of healthcare professionals in providing clear communication and support are critical areas that warrant further exploration. The success of the method depends on proper training for healthcare providers and commitment to the brace-wearing protocol by families [9]. Also, the socio-economic status of parents seems to be an important criterion for compliance [10]. Moreover, the literature now recognizes the importance of a continuous care pathway that ensures coordinated support from diagnosis through long-term follow-up, fostering holistic management for both the child and their family [11,12].

In France, another non-surgical treatment method is still fairly widespread: correction by means of physical therapy sessions, known as the functional or “French” method (FM) [13]. Since this method is more restrictive for the organization of family life, more and more families are opting for corrective treatment using the casting method [14]. Regarding PM, families often report difficulty in finding a practitioner trained in this type of treatment, and frustration at not feeling listened to when expressing their choice of treatment, which is regrettable given the high compliance rate among the families who participate in this method. The French method is known for being more expensive overall [10,15], even though no proper study has assessed these costs from a societal perspective in order to figure out expenses for different payers, and among them families. Faced with this problem, and unable to find any evidence after an exhaustive review of the literature, we set out to compare the two methods, to optimize treatment of idiopathic clubfoot, with medical and social criteria as a priority.

### 1.2. Objective

The first objective was to conduct a descriptive medico-economic and medico-social evaluation of two methods of treatment of clubfoot, the Ponseti and the functional treatment, during the first two years of life.

## 2. Materials and Methods

### 2.1. Study Design and Reporting Standards

This is a prospective, multicentric, non-interventional study of a consecutive series of patients who were treated in four pediatric university hospitals in France. All patients who met the following selection criteria: (a) newborns presenting with uni- or bilateral idiopathic clubfoot under the age of one month, (b) without receiving any previous treatment and (c) whose parents were able to express their agreement and had coverage by the national health insurance system, were included in the study. The research protocol was approved by two national ethics committees: CPP (Comité de Protection des Personnes, Ile de France IV) and CNIL (Comission Nationale de l’Informatique et des Libertés). Funding was provided by the French Health Ministry by a research grant [AOR12060]. All parents and legal guardians were informed and their consent to participate was obtained under the investigator’s responsibility.

We conducted a longitudinal study with a 24-month time horizon. This duration was selected to align with the framework of the institutional funding grant while ensuring a comprehensive tracking of the most resource-intensive phases of both protocols, thereby capturing the primary direct medical costs and initial clinical outcomes. Randomization was precluded by strong clinical and parental preferences for specific treatment modalities, raising ethical concerns regarding the restriction of family choice. Furthermore, the functional French method requires specialized physical therapists who were unavailable in centers exclusively offering the Ponseti method. Due to the anticipation of minimal or non-overlapping distributions between the two cohorts—as parents opting for the Ponseti method would systematically decline the functional treatment and vice versa—a propensity score matching approach was not pursued.

To ensure methodological transparency, the health economic evaluation component was prepared in accordance with the Consolidated Health Economic Evaluation Reporting Standards (CHEERS) 2022 statement [16] while the qualitative reporting of the medico-social assessment adhered to the COREQ statement [17] (see Supplementary Materials, Figures S1 and S2).

### 2.2. Population

#### 2.2.1. Medico-Economic Population

We included children meeting the inclusion criteria, who were born with unilateral or bilateral clubfoot, without other associated pathologies, aged one month or less and who had not received any treatment for this condition at the time of inclusion in the study.

#### 2.2.2. Medico-Social Sample

The study involved a total of 32 interviews, conducted using different methods and with different groups of participants. Parents were contacted by email and recruited on a voluntary basis. A semi-structured interview guide was developed to explore different aspects of caring for their child, including prenatal diagnosis, information-seeking processes, relationships with parents and healthcare professionals, as well as the challenges and resources associated with everyday life. The interview guide provided a flexible framework for the discussions, allowing the researcher to adapt the questions and follow-up prompts to each participant’s perspective and experiences. The initial interviews took place at the participants’ family homes and served as a basis for making adjustments to the interview guide as needed.

Sixteen interviews (approximately 1 h each) were conducted with parents from children included in the quantitative cohort, including 7 whose children were treated with the functional method and 9 with the Ponseti method. Among these, 11 interviews were conducted with mothers only, 1 with a father only, and the remaining 4 with both parents. The children were between 4 and 5 years old at the time of the interview, but had received treatment within the first year after birth. The majority of families interviewed came from middle or low socio-economic backgrounds. The interviews took place during the follow-up period.

Additionally, 16 telephone interviews (approximately 45 min each) were conducted with physicians at various orthopedic centers across France.

### 2.3. Intervention Strategies

#### 2.3.1. Ponseti Method

The Ponseti method consists of correcting the foot deviation by means of long-leg casts with the knee bent at 90°, which are changed once a week. Once the calcaneopedial block has been derotated, if residual equinus deformity of the hindfoot remains, a percutaneous tenotomy of the Achilles tendon is indicated. After the tenotomy, a long-leg cast is applied for three weeks. At the end of this period, a Denis-Browne type splint is started to maintain the correction. Current recommendations establish that if the foot correction is maintained, the splint can be discontinued at the age of four. Long-term follow-up is advised until maturity in order to make an early diagnosis of relapses.

#### 2.3.2. Functional Treatment

The functional treatment consists of a gradual correction of the deformity, with daily physical therapy sessions until an adequate correction is achieved. Manipulations are performed by a physical therapist and, between sessions, the correction obtained is maintained by a strapping, a molded splint or a soft cast. Once correction of the foot has been achieved, physical therapy sessions can be carried out twice or three times a week, and a night splint tends to be used. In the event of persistent isolated equinus, an Achilles tenotomy is performed, and can be followed by immobilization in a long-leg cast for four weeks.

### 2.4. Clinical Outcomes

The data collected were socio-demographic (gender, age), clinical data (weight at birth, Dimeglio score from birth to end of follow-up, pathology-related hospitalizations or surgical interventions). The principal clinical outcome was the Dimeglio score at two years follow-up, assessed in an open manner (unblinded) by the surgeon in charge of the treatment.

### 2.5. Economic Evaluation

We included direct medical costs (hospitalizations, consultations, splints, various procedures), direct non-medical costs (daily allowances for informal caregivers), indirect costs (loss of productivity) and medical costs for parents (out-of-pocket expenses). Care consumption data were collected in the CRF at the end of the study.

Costs were estimated for all patients included and valued in euros in 2025 with a 2.5% discount as recommended in France [18].

Hospitalizations (mainly for tenotomies) were valued using the National cost study with common methodology (ENCC) 2019. Procedures such as X-rays and tenotomies, as well as physiotherapy sessions and splints, were valued using the health insurance tariff for the procedure or product. The cost of a consultation was valued using the salary of an Associate Professor with added contributions, with an estimated consultation time of 15 min. Cost details are presented in Supplementary Data, Table S1.

Out-of-pocket (OOP) expenses corresponded to the total amount paid by parents for purchases related to their child’s clubfoot treatment, which are not covered by health insurance or a mutual insurance company.

The cost of days off work for informal caregivers (parents) was calculated according to the following:

Cost of work absence for informal caregivers = daily indemnity * number of days off.

The time spent by families caring for their child was valued using the human capital method (HCM). The loss of production due to days off was estimated on the basis of the average contribution to the average French wage. As for informal care costs, we considered that only one parent had to take a day off, and that in the case of bilateral clubfeet, procedures on both feet were carried out at the same time. The same number of days of absence was therefore counted for bilateral cases. Consultation counted for half a day and tenotomy or hospitalization for a whole day.

Given the multiple stakeholders (children’s parents, caregivers, hospitals), we undertook a cost-consequence analysis which reports the multidimensional nature of costs and treatment [19]. The perspective chosen was societal, and the time horizon was 24 months.

Costs were described by type of cost and by family, for one or two affected feet. The two variables studied as ‘consequences’ were the Dimeglio score at 24 months and the percentage of children reaching the “benign foot” stage on the Dimeglio score (score < 5) at 24 months.

### 2.6. Social Evaluation

Outcomes were 1. From the parents’ point of view: the social acceptability of therapeutic management, as well as motivations and barriers to treatment; 2. From the professionals’ point of view: the social acceptability of therapeutic management, and personal arguments in favor of the choice of treatment.

The study involved two main methods of data collection:

- Semi-structured face-to-face interviews: A sociologist trained in public health approach conducted these interviews in the homes of families affected by congenital clubfoot. This method allows for in-depth exploration of the participants’ experiences and perspectives.
- Telephone interviews: A public health researcher conducted these interviews with professionals at various pediatric orthopedic centers in France. This method was chosen in part due to logistical and budgetary constraints, while still enabling access to valuable insights from experts who were geographically dispersed.

### 2.7. Analysis

#### 2.7.1. Medico-Economic Analysis

##### Missing Data

No missing data were observed for baseline characteristics. During follow-up, missing data occurred for both longitudinal Dimeglio scores and resource-use variables. At 24 months, the proportion of missing Dimeglio scores ranged from 22.0% to 69.2% across treatment groups and according to whether the condition was unilateral or bilateral (Supplementary Data, Table S2). For resource-use variables, missing data concerned splint type, out-of-pocket costs, and whether a medical consultation occurred at each visit. The proportion of missing data was below 20% for most resource-use variables and visits. Higher proportions were observed at some later visits, particularly for out-of-pocket costs and splint type, reaching 69.2% and 87.5%, respectively, at specific visits (Supplementary Data, Table S2).

Missingness in both the Dimeglio score and resource-use variables was not significantly associated with treatment allocation. Data were assumed to be missing at random (MAR). Missing values were imputed by multiple imputation using chained equations (MICE), performed separately within each treatment group.

Missing Dimeglio scores were imputed by multilevel multiple imputation using chained equations (MICE), performed separately within each treatment group, modeling the score as a count variable following a negative binomial distribution given its longitudinal structure. Predictors included sex, age at inclusion, antenatal diagnosis, pathology group, gestational term, birth weight, delivery mode, and center, together with previously observed Dimeglio scores at earlier visits, consistent with the temporal ordering of data collection. Care consumption (by cost category) was imputed using mean matching, with predictors including sex, age at inclusion, antenatal diagnosis, pathology group, gestational term, birth weight, delivery mode, and center, and the other care consumption categories. Because costs and clinical outcomes are typically correlated, the Dimeglio score and care consumption were included as mutual predictors to preserve this association and avoid bias in the cost-consequence estimates.

Imputation was performed in R (version 4.2.1) using the “mice” and “countimp” packages, generating 20 imputed datasets per treatment group. Estimates were pooled according to Rubin’s rules [20]. Confidence intervals for means, percentages, and their differences were computed using a bootstrap procedure with 1000 resamples, applied within each of the 20 imputed datasets, yielding a combined set of 20,000 bootstrap estimates from which the 95% confidence interval was derived using the percentile method [21]. The plausibility of imputed values and their stability across imputations were assessed graphically (Supplementary Data, Figure S3).

To assess the robustness of the imputed estimates to departures from the MAR assumption, we conducted two sensitivity analyses at 24 months for each treatment group, applied to both the continuous Dimeglio score and the derived binary outcome (benign foot, Dimeglio score < 5): a delta-adjustment, shifting the imputed values of originally missing observations by δ = −4 to +4 points to simulate departures from MAR in either direction, and worst-case/best-case bounding, assigning missing observations the least and most favorable possible score. Full results are presented in Table S3 and Figure S4.

##### Base-Case Analysis

The analysis was conducted separately for patients with bilateral and unilateral clubfoot.

##### Subgroup Analysis

Subgroup analyses were performed based on the severity of clubfoot at inclusion. The two distinct groups considered were patients with a very severe clubfoot and those with moderate or severe clubfoot. For individuals with bilateral clubfoot, those with at least one foot classified as very severe were grouped under ‘very severe clubfoot’.

##### Sensitivity Analysis

A one-way deterministic sensitivity analysis was performed to identify the main factors influencing costs and to account for uncertainty in the base-case analysis. Test and procedure tariffs (imaging, tenotomies, casting, physiotherapy sessions and medical consultations), splint tariffs, daily indemnities and the average French wage were varied by upper and lower bounds of 10%. The results of the deterministic sensitivity analysis were expressed in a tornado diagram.

To verify the robustness of the findings, scenario analyses were conducted to examine how different assumptions could influence results: scenario 1: no imputation was performed for missing data, only patients who had completed a 24-month follow-up were included in the analysis; scenario 2: all tenotomies were considered performed in a day hospital setting without requiring a hospital stay; scenario 3: all patients’ feet treated with the functional method were braced with the least expensive brace; scenario 4: all patients’ feet treated with the functional method were braced with the most expensive brace.

#### 2.7.2. Medico-Social Analysis

Data analysis was facilitated through the use of NVivo 10 software for family interviews, while the professional interviews were analyzed manually. A total of 16 parents of children treated for congenital clubfoot participated in this research, with recruitment continuing until data saturation was reached, that is, until new interviews no longer yielded novel insights and the information collected became redundant. Interviews were coded using NVivo10, drawing on a thematic tree composed of analytical categories collaboratively defined by the research team: challenge of diagnosis, communication and anticipation, treatment choice, the role of healthcare teams and social networks, and the perceived benefits and limitations. Thematic coding was carried out by at least two researchers in order to guarantee the reliability of the data collected from professionals and families, with a view to cross-referencing. Throughout the analytical process, the team maintained a reflexive stance, ensuring that coding remained grounded in parents’ own words and experiences rather than in pre-existing clinical assumptions.

The final analysis was conducted collaboratively by the entire multidisciplinary research team, fostering a comprehensive and shared understanding of the data. This multifaceted approach enabled a nuanced exploration of the experiences and perspectives of both families and healthcare professionals, thereby enriching the depth and quality of the qualitative findings.

## 3. Results

### 3.1. Medico-Economic Evaluation

#### 3.1.1. Population and Clinical Outcomes

**Figure 1:**
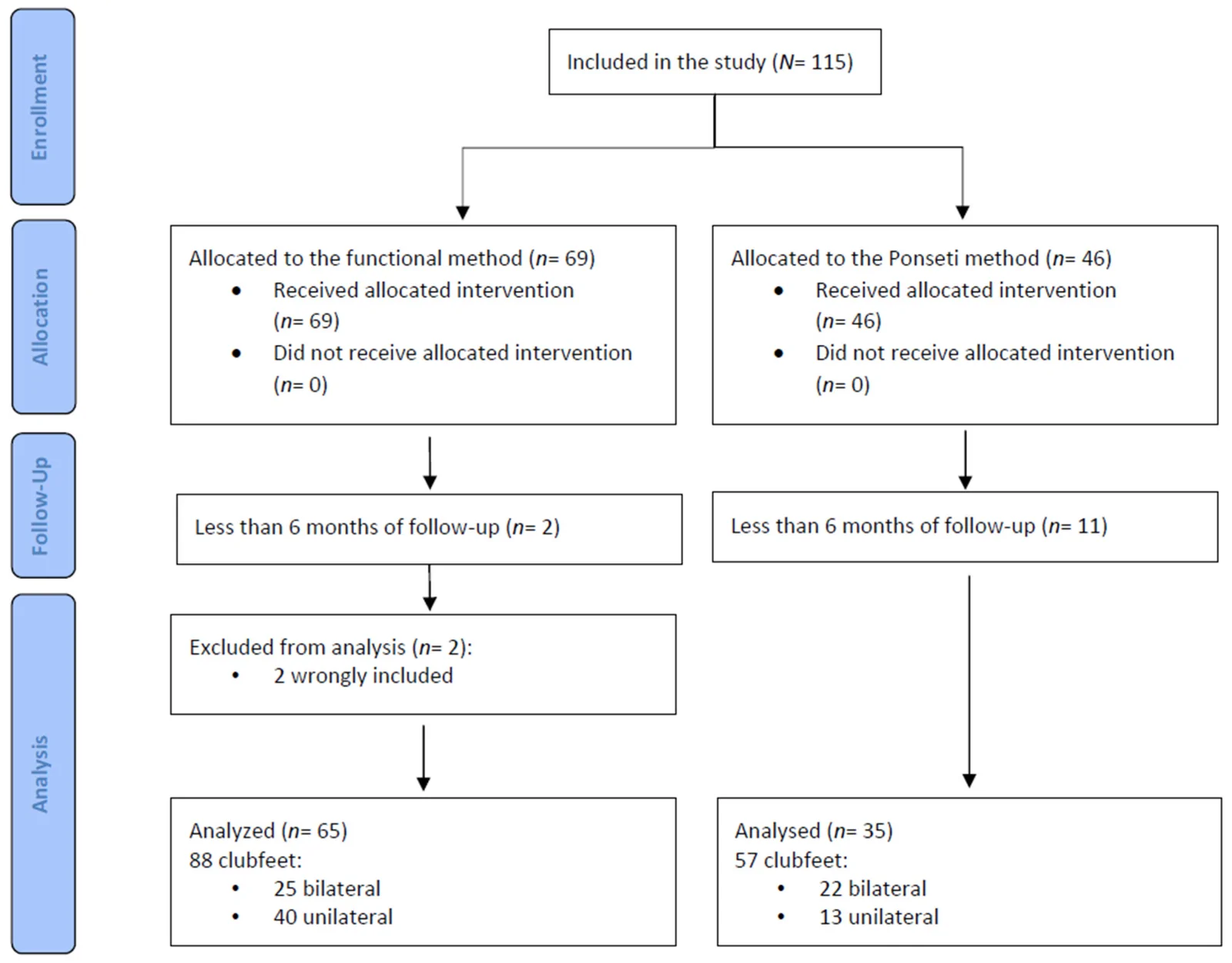
Flowchart (medico-economic analysis).

A total of 115 patients were enrolled in the study from January 2014 to December 2016 (46 for the Ponseti method and 69 for the functional method) across four pediatric university hospitals, with one of them practicing both methods, one exclusively the functional method, and two exclusively the Ponseti method. Out of these, 13 patients were excluded due to less than 6 months of follow-up, and two were wrongly included and thus were excluded from the analysis (see Figure 1).

In total, 100 patients meeting the inclusion criteria were included in the economic analysis (35 for the Ponseti method and 65 for the functional method), corresponding to 145 clubfeet (57 for the Ponseti method and 88 for the functional method). Table 1 presents the patient characteristics for both groups. No patients underwent extensive surgery during follow-up. At the last follow-up (median 18.1 [13.8; 23.3] months of life), 97% of feet in the PM group and 96% in the FM group obtained a Dimeglio score < 5/20.

#### 3.1.2. Cost-Consequences Analysis

##### Base-Case Analysis

The percentages of patients with benign clubfoot at 24 months were over 90% in both groups. The Functional method was associated with higher direct medical costs, direct non-medical costs, and indirect costs. In contrast, out-of-pocket expenses for parents were higher for the Ponseti method (Table 2 and Table 3).

##### Subgroup Analysis

**Figure 2:**
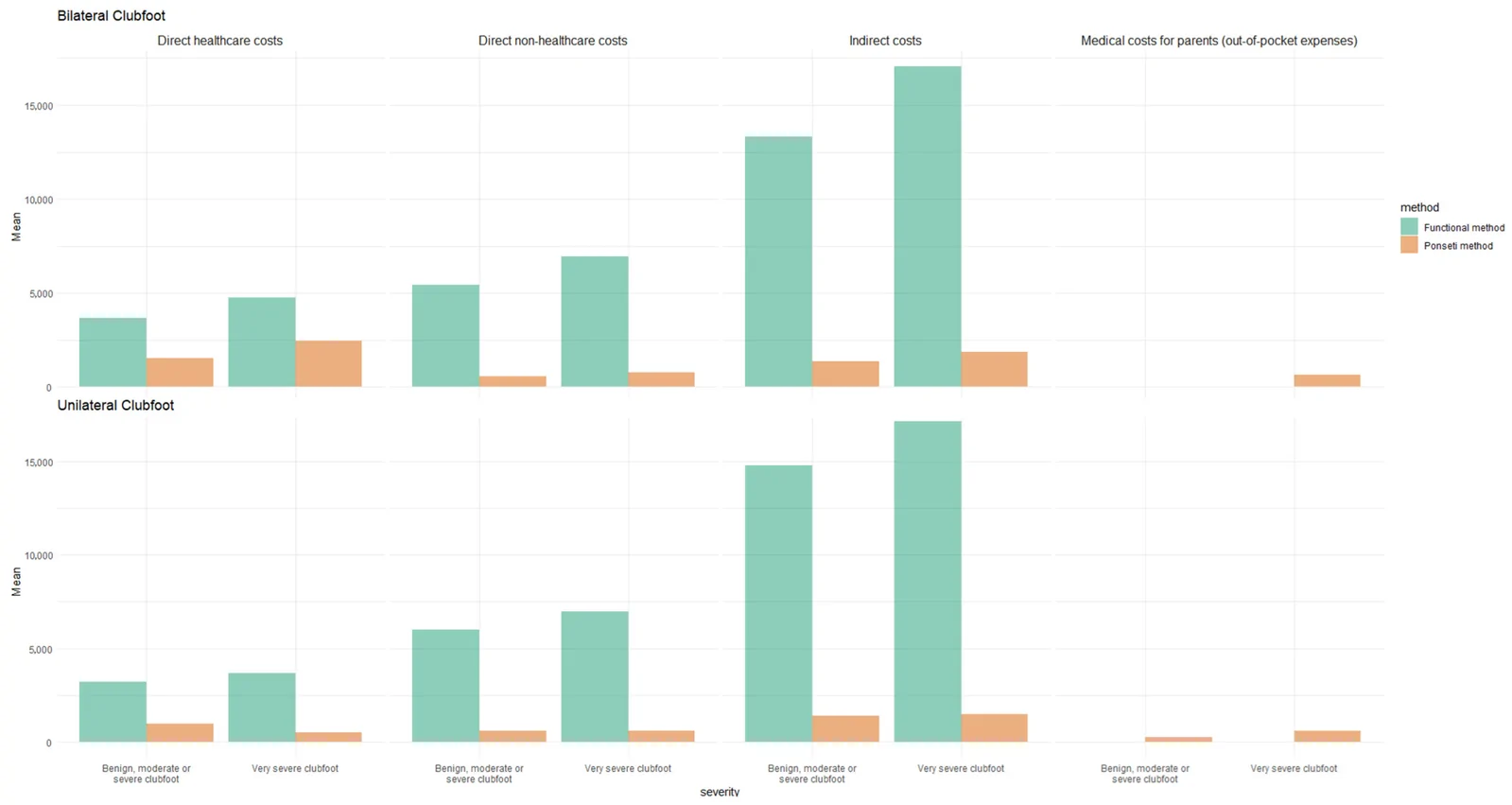
Subgroup analysis of costs.

Figure 2 illustrates the different types of costs by severity and treatment method, and the results are consistent with the base-case analysis. Direct medical costs, direct non-medical costs, indirect costs, and out-of-pocket expenses are higher in patients with a very severe clubfoot at baseline. Consequences for clubfeet, by severity at inclusion, are presented in Supplementary Data, Tables S4 and S5.

##### Sensitivity Analysis (SA): One-Way Deterministic SA

**Figure 3:**
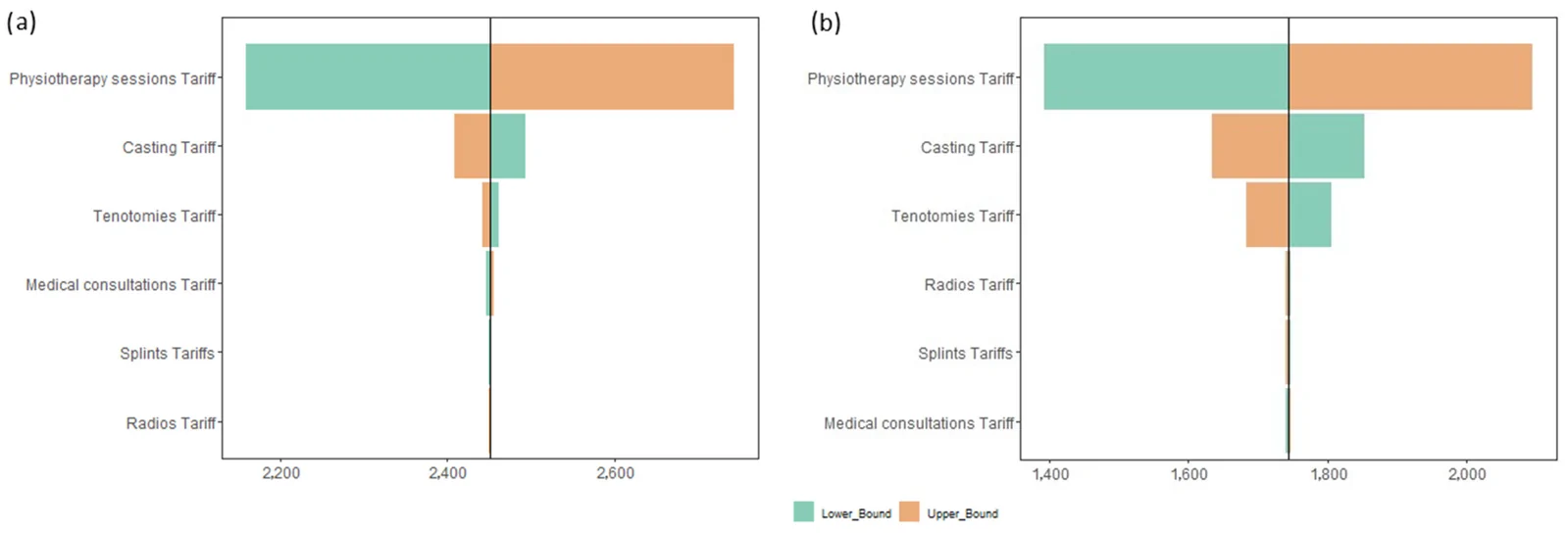
Tornado diagram of direct medical costs (Functional method—Ponseti method). (**a**) Unilateral clubfoot; (**b**) bilateral clubfoot.

Based on the one-way deterministic analysis, the three factors that most influence direct medical costs are consistent for both unilateral and bilateral clubfoot (Annex Figure 3): physiotherapy sessions, casting and tenotomies tariffs.

**Figure 4:**
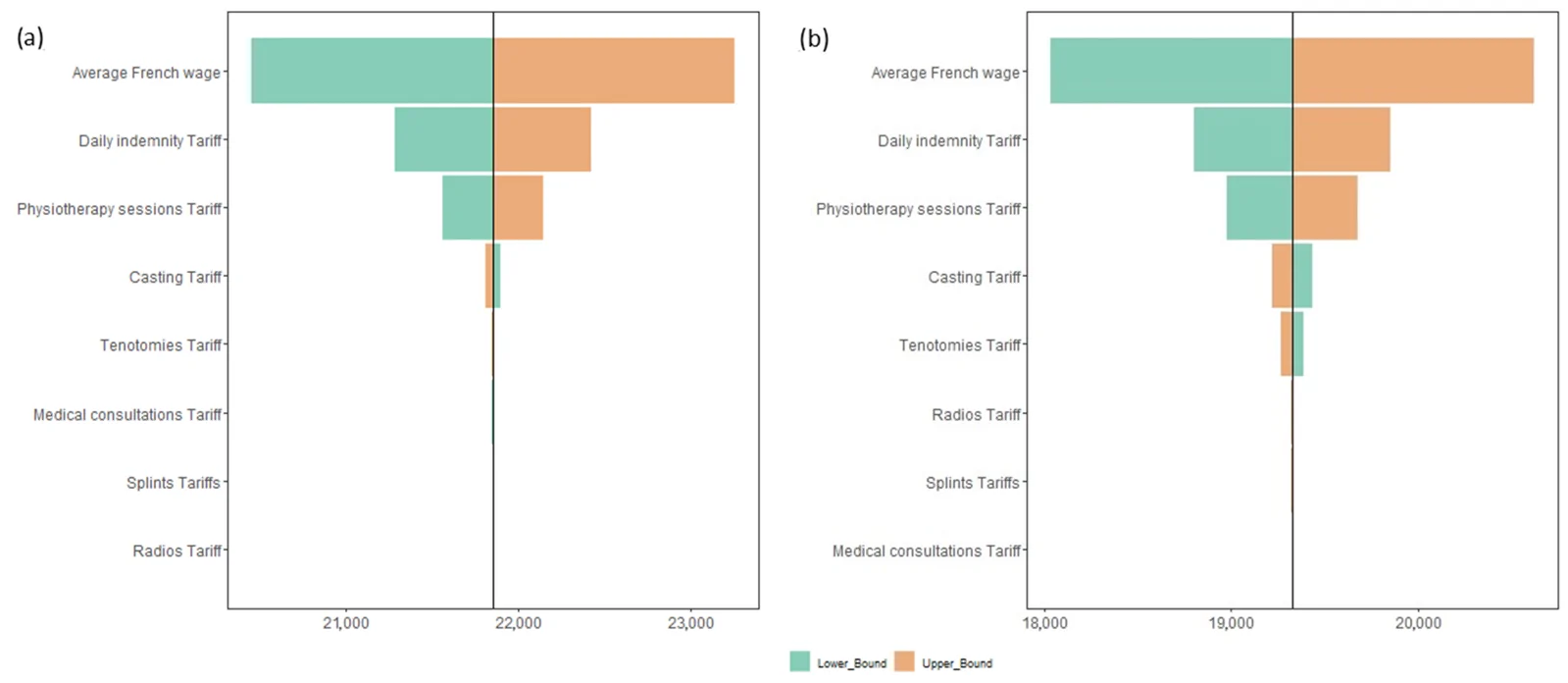
Tornado diagram of total costs (Functional method—Ponseti method). (**a**) Unilateral clubfoot; (**b**) bilateral clubfoot.

When all cost components are taken into account (Figure 4), the SA highlighted lost productivity (measured by average wages) as the factor whose variation has the greatest impact on the difference in costs between the two methods. In any case, the variation in costs did not alter the observed lower costs of the Ponseti method compared to the functional method.

##### Scenario Analysis SA

**Figure 5:**
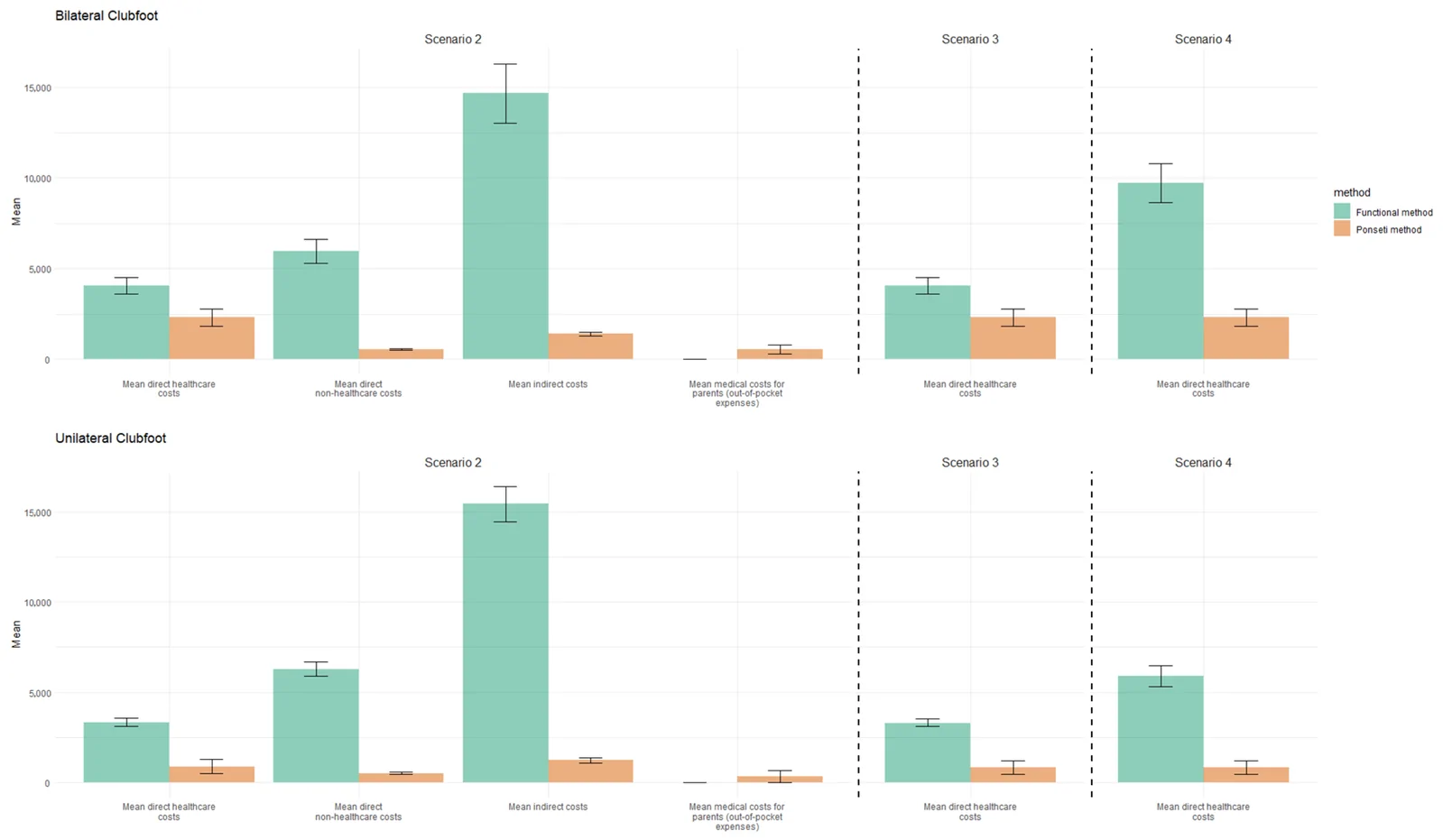
Comparison of cost details for both methods by scenarios (Scenarios 2 to 4) for uni- and bilateral impairments. Range-lines: standard deviation.

Results of the four scenarios (Figure 5) were broadly consistent with those of the base-case analysis. They demonstrated the robustness of the cost analysis in response to changes in assumptions. The results of scenario analysis 1 are presented in Table 4 and Table 5.

### 3.2. Medico-Socio Analysis

#### 3.2.1. The Challenge of Diagnosis

Receiving a diagnosis of congenital clubfoot can trigger significant anxiety in parents, primarily due to fears of more serious underlying conditions. Benoît shared his experience:

“He tried to reassure us, but as soon as we heard there might be a chromosomal issue, we feared the worst. We immediately thought of Down syndrome [also known as trisomy 21], and that was terrifying.”

(Benoit, 35 yo, middle class)

Lucie echoed this sentiment, explaining:

“Clubfoot can either be isolated or linked to another condition, and we were really scared it could be spina bifida [birth defect that can cause physical and nerve problems, like difficulty walking or bladder issues]. The implications for treatment and future life are entirely different in those cases.”

(Lucie, 38 yo, upper class)

Feelings of guilt, especially among mothers, were a recurring theme in the testimonies. Benoît (35 yo, middle class) added, “It was particularly hard on my wife. Since she was carrying the baby, she felt responsible.”

The moment of discovering the malformation is pivotal for many parents. Kader recalled the shock:

“We found out on the day of the birth. I was astonished because all the tests we had requested were normal. I was surprised they hadn’t noticed it earlier.”

(Kader, 40 yo, low class)

In contrast, Benoît felt fortunate to have been informed early on:

“It’s a huge advantage to know before birth. When you find out at birth, it’s overwhelming. We had several months to process the information, gather resources, and meet with the doctor in advance.”

(Benoit, 35 yo, middle class)

The announcement of a congenital clubfoot diagnosis often triggers intense emotional reactions in parents, marked by anxiety, guilt, and fear of more severe conditions. Early detection can play a crucial role in helping families prepare emotionally and practically, while late discovery may lead to feelings of shock and unpreparedness. These testimonies highlight the need for sensitive communication and support during the diagnostic process.

#### 3.2.2. The Importance of Communication and Anticipation

The manner in which the diagnosis is communicated significantly impacts parents. Lucie (38 yo, upper class) expressed her desire to avoid reliving the anxiety tied to this announcement: “We specified in the birth plan that we didn’t want them to mention the feet at birth. We wanted to welcome the baby, not focus on the feet.” explained Joel (39 yo, middle class).

Nicole lamented a lack of clarity in the explanations given:

“I think there’s progress to be made at the hospital. At the very least, explaining what clubfoot is and what it entails would help. It was more like, ‘Make an appointment with the physiotherapist when you leave, figure it out yourselves.’ ”

(Nicole, 37 yo, low class)

The way a clubfoot diagnosis is delivered deeply shapes parental experience. Clear, empathetic communication can ease initial distress, while vague or abrupt announcements may intensify confusion and anxiety. Parents emphasized the importance of preserving the emotional integrity of birth moments and receiving adequate information to navigate the condition with confidence.

#### 3.2.3. Treatment Choice: Families’ Perspectives

In several cases, parents reported feeling steered toward a specific treatment method without being fully informed of alternatives. Nicole (37 yo, low class) notes: “We were offered only the physiotherapy method without being presented with other options.”

For others, the trust established with the medical team played a decisive role. Lucie recounted: “He reassured us, saying our child would have a normal life…” The perceived well-being of the child also weighed heavily on these decisions. Rachel (30 yo, low class) explained: “I wanted her to be stimulated; I didn’t want her to be bedridden with a cast all the time.” Similarly, Joel (39 yo, middle class) mentioned choosing a “more flexible” method that seemed more comfortable for the child.

Practical considerations and lifestyle constraints also shaped parental choices. Marlène (39 yo, low class) stated: “I absolutely could not travel to physiotherapy daily.” Fanny (41 yo, middle class) adds: “Given our busy work schedules, it became clear that this method would be the most feasible for us.” These realities often made the Ponseti method, with its less intensive follow-up, more attractive.

Parental decisions regarding treatment were influenced by a combination of medical guidance, concern for the child’s development and comfort, and logistical realities. While some parents felt a lack of autonomy in the decision-making process, others relied on trust in healthcare professionals. The balance between medical advice, everyday feasibility, and perceived quality of life for the child emerged as central to their choices.

#### 3.2.4. The Crucial Role of Healthcare Team and Social Networks

The support of the healthcare team is essential for families dealing with congenital clubfoot. One mother describes the mental load involved in daily care routines: “She has physiotherapy at 2 p.m., so I need to finish her bath by 12:30. I remove the brace by 11:30 for an hour of freedom… we can talk about mental workload!” (Rachel, 30 yo, low class).

Physiotherapists often become key points of contact for families, providing continuity and reassurance. One parent explained:

“The physiotherapist acts as a privileged contact for the family. Doctors have less time to engage in conversation, while the physiotherapist provides reassurance. I often ask her to revisit discussions that feel rushed, as the information can be confusing.”

Beyond clinical support, social media networks play a vital role in offering both emotional comfort and practical advice. One parent reflected: “This group has acted as a psychological support unit for us.”

These communities allow for creative resource sharing, such as modified clothing to accommodate braces. Marlène noted: “There are exchanges of pajamas cut specifically for braces. Some grandmothers knit covers and winter overshoes to keep the children warm.” (Marlène, 39 yo, low class).

This solidarity fosters a strong sense of belonging and mutual aid among families, helping them to better navigate the everyday challenges of treatment. Frequent interactions with healthcare professionals, combined with the informal emotional support of online communities, greatly enrich the parenting experience.

As single mothers like Rachel may face additional challenges, strong support networks proved all the more essential. As she shares: “Fortunately, my mother was there to help me.”

The journey through clubfoot treatment is shaped not only by medical care but also by emotional, logistical, and social factors. Healthcare teams (including physiotherapists, nurses, etc.) offer vital, ongoing support and reassurance, often filling in the communication gaps left by busy doctors. Meanwhile, online communities and family networks provide practical help and a sense of solidarity, especially valuable for parents facing the journey alone. These interconnected forms of support ease the burden and empower families in their caregiving roles.

#### 3.2.5. Perceived Benefits and Limitations

##### Benefits

The Ponseti method is renowned for its effectiveness, significantly lowering the need for surgical interventions. A practitioner observes: “The results are clear: the rate of extensive surgeries is extremely low with the Ponseti method.”

The method is perceived as less burdensome for families. A physician comments: “This method is much easier to endure than going to physiotherapy multiple times a week [as prescribed with the functional method].” This alleviates logistical stress for parents, particularly those living far from care.

Many families accept this method, often already informed about its efficacy. A practitioner notes, “I have never encountered a refusal to implement the treatment.”

Praised for its clinical effectiveness and practical convenience, the Ponseti method is generally well received by both practitioners and families. Its ability to minimize the need for surgery and reduce treatment burden makes it an attractive and accessible option, especially for those facing logistical constraints, in contrast to the functional method, which requires frequent physiotherapy sessions over an extended period. Nevertheless, some parents seemed to feel comforted by close monitoring by physiotherapist in FM approach.

##### Limitations

A major point of criticism is the lack of options. A professional explains: “There is no choice. We merely explain why we have chosen this method.” This can lead to frustration and a sense of helplessness, especially among single mothers who have to deal with care alone.

The success of the treatment relies heavily on family involvement. Practitioners note that compliance is crucial, especially during the brace-wearing phase.

The costs associated with orthopedic devices pose a significant challenge. Families may face out-of-pocket expenses that create barriers, particularly for those in precarious situations. One professional points out: “Costs can reach up to €400, often borne by the families themselves.”

Despite its clinical success, the clubfoot treatment raises concerns around autonomy, parental burden, and financial accessibility. The lack of treatment choice and high expectations for family involvement can leave some parents—especially single or vulnerable ones—feeling unsupported. In addition, as highlighted by the economic evaluation, the cost of orthopedic equipment remains a critical barrier that may hinder equitable access to care.

## 4. Discussion

We present the results of an observational comparison of two methods currently used for the treatment of clubfoot, functional and Ponseti. Our results suggest that, whatever the treatment method, clinical outcomes at 24 months did not differ whether the pathology was uni- or bilateral, very severe or not, with the proportion of children reaching this “benign foot” score (Dimeglio < 5) at 24 months being over 90% in each group. These findings are consistent with the fact that only compliance with wearing the splints was reported as a predictor of treatment efficacy, and not the presentation at birth.

Regarding costs, in the French context, the functional method was much more costly than the Ponseti method, with a ratio of 1/4 to 1/10, except for the parents’ out-of-pocket expenses, for which the trend was reversed. However, it seemed that in addition to sharing knowledge, the Ponseti network also creates a second-hand market that can help alleviate additional expenses for these families. All different scenarios of sensitivity analysis supported the base-case analysis, also pointing out physiotherapy-session, casting, and tenotomies tariffs as main items among direct medical costs. However, with regard to total cost, the main contributor was the loss of productivity.

Our findings regarding the higher cost of FM compared to PM were consistent with the international literature, which describes the Ponseti method as the easiest and least expensive to implement, particularly in developing countries [8].

Although observed clinical outcomes at 24 months appeared similar between treatment pathways, the observational design and wide confidence intervals of this study preclude any conclusion of clinical equivalence. Other studies following clubfoot evolution over five years [22] suggest that 24 months may be insufficient to establish comparative clinical effectiveness, and our short-term findings should be interpreted as descriptive rather than conclusive.

The main limitations of this analysis are that data pairing was not performed, making comparative analysis impossible, as well as the large amount of missing data, associated with the length of the study and recruitment problems. While imputation is an interesting tool for compensating for missing data, it does not replace a good-quality database. This concern was most pronounced in the unilateral Ponseti group (4 of 13 patients observed at 24 months), where complete-case and imputed benign-foot proportions differed substantially (75.0% vs. 92.3%); sensitivity analyses assessing departures from the missing-at-random assumption confirmed that estimates for the other three groups were robust, but indicated that this specific estimate should be interpreted with caution given the small number of observed patients (Supplementary Data, Table S3, Figure S4). Also, transport was not taken into account, due to the lack of information on family location.

Finally, biases, such as the fact that treatment is center-dependent, and the heterogeneity of follow-up from one center to another, some centers keeping children for up to 24 months, while others stopped follow-up when children reached a score under five, prevent us from reaching a conclusion on a superior treatment.

This is where sociological analysis can provide further insights regarding a more enlightened choice of method by families and pediatricians. Overall, the testimonies of families underscored the importance of clear and empathetic communication from healthcare providers to reassure parents and guide them in their treatment decisions. Although the choice is often influenced by professionals, the trust and active engagement of families are essential for adherence to treatment and its long-term success. While the FM approach may provide parents with a feeling of reassurance and support by the physiotherapist, the Ponseti method represents a more convenient treatment for families, combining effectiveness with a focus on the child’s well-being. Also, the fact that Ponseti families have OOP expenses is balanced out by the support they receive from the network of other families. However, it requires ongoing support (both emotional and practical) to ensure optimal care and a serene healthcare journey. Supporting single mothers in this process is crucial, as it reduces mental burdens and enhances the care experience for the entire family. More broadly, offering targeted support contributes to reducing social health inequalities by addressing the specific challenges faced by families in vulnerable situations.

## 5. Conclusions

Our results suggest that there may be no clinical differences between the two treatments in the short term, although clinical results were not sufficient to favor the use of either method. In the light of the results of the medico-economic and sociological aspects of the study, it would appear that, in France, even if the Ponseti method stood out as the most manageable for parents and healthcare providers, and the most economical for the healthcare system, its cost for families is not negligible, whereas FM can be highly demanding but allows children to remain in regular contact with a professional at no out-of-pocket cost. Parents need to be provided with relevant information on the various treatment options, to offer them the best possible support and to respect the particularities of their socio-economic environment in order to achieve the best degree of compliance.

## Figures and Tables

**Table 1:** Base-case patients’ characteristics.

	Functional Method	Ponseti Method	Overall
	(*n* = 65)	(*n* = 35)	(*N* = 100)
Gender. *n* (%)			
Male	43 (66.2%)	27 (77.1%)	70 (70.0%)
Female	22 (33.8%)	8 (22.9%)	30 (30.0%)
Age in days			
Median (Q1; Q3)	5.0 (3.0; 10.0)	11.0 (7.0; 14.0)	7.0 (4.0; 12.5)
Min; Max	0.0; 29.0	2.0; 28.0	0.0; 29.0
Antenatal diagnostic, *n* (%)			
No	13 (20.3%)	10 (28.6%)	23 (23.2%)
Yes	51 (79.7%)	25 (71.4%)	76 (76.8%)
Affected side, n (%)			
Unilateral	40 (61.5%)	13 (37.1%)	53 (53.0%)
Bilateral	25 (38.5%)	22 (62.9%)	47 (47.0%)
Mean Dimeglio score at inclusion, mean (95% CI)			
Unilateral	12.7 (11.8–13.6)	13.0 (11.6–14.4)	12.8 (11.9; 13.5)
Bilateral			
Left foot	11.5 (9.8–13.2)	15.3 (14.4–16.2)	13.2 (12.2; 14.3)
Right foot	12.5 (11.0–14.0)	15.2 (14.2–16.2)	13.6 (12.6; 14.6)

**Table 2:** Costs and outcomes for the base-case analysis (unilateral clubfoot).

	Functional Method	Ponseti Method	Incremental (Functional—Ponseti)
	(*n* = 40)	(*n* = 13)	
Mean Dimeglio score at 24 months (95% CI)	1.8 (1.3; 2.3)	1.1 (0.2; 2.2)	0.7 (−0.5; 1.7)
Percentage of patients with a benign clubfoot at 24 months (95% CI)	92.3 (84.0; 100.0)	92.3 (77.8; 100.0)	0.0 (−14.6; 18.2)
Mean direct medical costs (95% CI)	3340 (3045; 3687)	889 (631; 1256)	2451 (1981; 2886)
Mean direct non- medical costs (95% CI)	6275 (5720; 6921)	576 (460; 739)	5699 (5127; 6345)
Mean indirect costs (95% CI)	15,441 (14,085; 17,017)	1416 (1131; 1808)	14,025 (12,617; 15,639)
Mean medical costs for parents (out-of-pocket expenses) (95% CI)	0 (0; 0)	322 (92; 642)	−322 (−642; −92)

**Table 3:** Costs and outcomes for the base-case analysis (bilateral clubfoot).

	Functional Method	Ponseti Method	Incremental (Functional—Ponseti)
	(*n* = 25)	(*n* = 22)	
Left foot			
Mean Dimeglio score at 24 months (95% CI)	1.0 (0.4; 1.7)	1.21 (0.71; 1.79)	−0.2 (−1.0; 0.7)
Percentage of patients with a benign clubfoot at 24 months (95% CI)	97.4 (88.0; 100.0)	95.2 (83.3; 100.0)	2.2 (−8.0; 12.7)
Right foot			
Mean Dimeglio score at 24 months (95% CI)	1.4 (0.8; 2.2)	1.1 (0.6; 1.8)	0.3 (−0.6; 1.3)
Percentage of patients with a benign clubfoot at 24 months (95% CI)	95.2 (84.0; 100.0)	94.6 (83.3; 100.0)	0.6 (−12.0; 12.8)
Mean direct medical costs (95% CI)	4061 (3595; 4539)	2318 (1911; 2796)	1746 (1096; 2377)
Mean direct non-medical costs (95% CI)	5968 (5299; 6645)	726 (599; 870)	5240 (4557; 5930)
Mean indirect costs (95% CI)	14,672 (13,010; 16,352)	1786 (1472; 2148)	12,896 (11,214; 14,593)
Mean medical costs for parents (out-of-pocket expenses) (95% CI)	1 (0; 3)	546 (332; 776)	−545 (−775; −335)

**Table 4:** Scenario analysis 1: non-imputed data (unilateral clubfoot).

	Functional Method	Ponseti Method
	(*n* = 31)	(*n* = 4)
Mean Dimeglio score at 24 months (95% CI)	2.1 (1.6; 2.6)	3.5 (3.0; 4.5)
Percentage of patients with a benign clubfoot at 24 months (95% CI)	90.3 (80.6; 100.0)	75.0 (25.0; 100.0)
Mean direct medical costs (95% CI)	2856 (2494; 3199)	682 (478; 952)
Mean direct non-medical costs (95% CI)	5371 (4637; 6042)	432 (337; 505)

**Table 5:** Scenario analysis 1: non-imputed data (bilateral clubfoot).

	Functional Method	Ponseti Method
	(*n* = 20)	(*n* = 12)
Left foot		
Mean Dimeglio score at 24 months (95% CI)	0.8 (0.3; 1.4)	2.2 (1.7; 2.8)
Percentage of patients with a benign clubfoot at 24 months (95% CI)	100 (100; 100)	92 (75; 100)
Right foot		
Mean Dimeglio score at 24 months (95% CI)	1.5 (0.8; 2.6)	2.0 (1.4; 2.8)
Percentage of patients with a benign clubfoot at 24 months (95% CI)	95 (85; 100)	92 (75; 100)
Mean direct medical costs (95% CI)	3703 (3036; 4305)	2207 (1787; 2705)
Mean direct non-medical costs (95% CI)	5423 (4515; 6251)	721 (508; 999)
Mean indirect costs (95% CI)	13,414 (11,184; 15,560)	1756 (1214; 2439)
Mean healthcare costs for parents (out-of-pocket expenses) (95% CI)	0 (0; 0)	601 (439; 769)

## Data Availability

This study was sponsored by the Assistance Publique—Hôpitaux de Paris (AP-HP); it received approval from the Ile-de-France IV Committee for the Protection of Persons (CPP) under reference number CPP2013/14NI and was authorized by the National Commission for Information Technology and Civil Liberties (CNIL) under No. ENRCNIL DR-2013-517. These authorizations strictly regulate the conditions for the use and sharing of individual data derived from this study and do not permit their public deposit or dissemination.

## Supplementary Materials

The following supporting information can be downloaded at: https://www.mdpi.com/article/10.3390/healthcare14203519/s1, Figure S1. CHEERS 2022 statement; Figure S2. QOREC statement; Figure S3. Diagnostics plausibility and stability of imputed values; Figure S4. Sensitivity tipping point analysis under missing-at-random assumption; Table S1: Unit costs and data sources for health care consumption; Table S2: Proportion of missing data; Table S3: Complete-case, multiply imputed, worst-case, and best-case estimates of benign-foot proportion at 24 months; Table S4: Sub-groups analysis: consequences for unilateral clubfeet, by severity at inclusion; Table S5: Sub-groups analysis: consequences for bilateral clubfeet, by severity at inclusion.

## Abbreviations

The following abbreviations are used in this manuscript:

HCM	Human Capital Method
MICE	Multiple Imputation by Chained Equations
OOP	Out-of-pocket
SA	Sensitivity analysis
