# Supplementary material for: Medico-Economic and Medico-Social Evaluation of Two Types of Treatment for Clubfoot in France: A Prospective Study Using a Mixed Methods Approach
Source: Healthcare (Basel). 2026 Oct 19;14(20):3519. doi: 10.3390/healthcare14203519 (PMC13704009; doi:10.3390/healthcare14203519)
Supplement: Supplementary file 1 [file healthcare-14-03519-s001.zip › healthcare-4440125-supplementary.pdf]

Supplementary Materials

Figure S1. CHEERS 2022 statement.

CHEERS 2022 Checklist

**Title**			
Title	1	Identify the study as an economic evaluation and specify the interventions being compared.	Title
**Abstract**			
Abstract	2	Provide a structured summary that highlights context, key methods, results, and alternative analyses.	Abstract
**Introduction**			
Background and objectives	3	Give the context for the study, the study question, and its practical relevance for decision making in policy or practice.	Introduction
Methods			
**Health economic analysis plan**	**4**	**Indicate whether a health economic analysis plan was developed and where available.**	**Not reported**
Study population	5	Describe characteristics of the study population (such as age range, demographics, socioeconomic, or clinical characteristics).	2.2.2. Medico-economic
Setting and location	6	Provide relevant contextual information that may influence findings.	2.1. Study design
Comparators	7	Describe the interventions or strategies being compared and why chosen.	2.3. Intervention strategies
Perspective	8	State the perspective(s) adopted by the study and why chosen.	2.5. Economic evaluation; paragraph 7
Time horizon	9	State the time horizon for the study and why appropriate.	2.5. Economic evaluation; paragraph 7

Discount rate	10	Report the discount rate(s) and reason chosen.	2.5. Economic evaluation; paragraph 1
Selection of outcomes	11	Describe what outcomes were used as the measure(s) of benefit(s) and harm(s).	2.4.2. Medico-economic
Measurement of outcomes	12	Describe how outcomes used to capture benefit(s) and harm(s) were measured.	2.4.2. Medico-economic
Valuation of outcomes	13	Describe the population and methods used to measure and value outcomes.	2.5. Economic evaluation
Measurement and valuation of resources and costs	14	Describe how costs were valued.	2.5. Economic evaluation
Currency, price date, and conversion	15	Report the dates of the estimated resource quantities and unit costs, plus the currency and year of conversion.	2.5. Economic evaluation + Table S1
Rationale and description of model	16	If modelling is used, describe in detail and why used. Report if the model is publicly available and where it can be accessed.	**NA**
Analytics and assumptions	17	Describe any methods for analysing or statistically transforming data, any extrapolation methods, and approaches for validating any model used.	2.7.2. Medico-economic
Characterising heterogeneity	18	Describe any methods used for estimating how the results of the study vary for subgroups.	2.7.2. Medico-economic; Subgroup's analysis
Characterising distributional effects	19	Describe how impacts are distributed across different individuals or adjustments made to reflect priority populations.	2.7.2. Medico-economic; Base-case analysis

Characterising uncertainty	20	Describe methods to characterise any sources of uncertainty in the analysis.	2.7.2. Medico-economic; Missing data; Sensitivity analysis
Approach to engagement with patients and others affected by the study	21	Describe any approaches to engage patients or service recipients, the general public, communities, or stakeholders (such as clinicians or payers) in the design of the study.	**NA**
**Results**			
Study parameters	22	Report all analytic inputs (such as values, ranges, references) including uncertainty or distributional assumptions.	3.1.2. Medico-economic
Summary of main results	23	Report the mean values for the main categories of costs and outcomes of interest and summarise them in the most appropriate overall measure.	3.3.1. Cost-consequences analysis; Base-case analysis; Sub-groups analysis
Effect of uncertainty	24	Describe how uncertainty about analytic judgments, inputs, or projections affect findings. Report the effect of choice of discount rate and time horizon, if applicable.	3.3.1. Cost-consequences analysis; Sensitivity analysis (SA): One-way deterministic SA; Scenario analysis SA
Effect of engagement with patients and others affected by the study	25	Report on any difference patient/service recipient, general public, community, or stakeholder involvement made to the approach or findings of the study	NA
Discussion			

Study findings, limitations, generalisability, and current knowledge	26	Report key findings, limitations, ethical or equity considerations not captured, and how these could affect patients, policy, or practice.	Discussion
Other relevant information			
Source of funding	27	Describe how the study was funded and any role of the funder in the identification, design, conduct, and reporting of the analysis	Funding
Conflicts of interest	28	Report authors conflicts of interest according to journal or International Committee of Medical Journal Editors requirements.	Conflicts of Interest

Husereau, D.; Drummond, M.; Augustovski, F.; de Bekker-Grob, E.; Briggs, A.H.; Carswell, C.; Caulley, L.; Chaiyakunapruk, N.; Greenberg, D.; Loder, E.; et al. Consolidated Health Economic Evaluation Reporting Standards 2022 (CHEERS 2022) Statement: Updated Reporting Guidance for Health Economic Evaluations. *MDM Policy Pract.* **2022**, *7*, 23814683211061096. https://doi.org/10.1177/23814683211061097

Figure S2. QOREC statement.

COREQ (COnsolidated criteria for REporting Qualitative research) Checklist

A checklist of items that should be included in reports of qualitative research. You must report the page number in your manuscript where you consider each of the items listed in this checklist. If you have not included this information, either revise your manuscript accordingly before submitting or note N/A.

Topic	Item No.	Guide Questions/Description	Reported on Page No.
**Domain 1: Research team and reflexivity**			
Personal characteristics			
Interviewer/facilitator	1	Which author/s conducted the interview or focus group?	p. 7
Credentials	2	What were the researcher's credentials? E.g. PhD, MD	N/A
Occupation	3	What was their occupation at the time of the study?	p.7
Gender	4	Was the researcher male or female?	N/A
Experience and training	5	What experience or training did the researcher have?	p.7
Relationship with participants			
Relationship established	6	Was a relationship established prior to study commencement?	N/A
Participant knowledge of the interviewer	7	What did the participants know about the researcher? e.g. personal goals, reasons for doing the research	N/A
Interviewer characteristics	8	What characteristics were reported about the inter viewer/facilitator? e.g. Bias, assumptions, reasons and interests in the research topic	N/A
**Domain 2: Study design**			
Theoretical framework			
Methodological orientation and Theory	9	What methodological orientation was stated to underpin the study? e.g. grounded theory, discourse analysis, ethnography, phenomenology, content analysis	p. 9
Participant selection			
Sampling	10	How were participants selected? e.g. purposive, convenience, consecutive, snowball	N/A
Method of approach	11	How were participants approached? e.g. face-to-face, telephone, mail, email	N/A
Sample size	12	How many participants were in the study?	p. 5
Non-participation	13	How many people refused to participate or dropped out? Reasons?	N/A
Setting			
Setting of data collection	14	Where was the data collected? e.g. home, clinic, workplace	p. 5
Presence of non-participants	15	Was anyone else present besides the participants and researchers?	N/A
Description of sample	16	What are the important characteristics of the sample? e.g. demographic data, date	p. 5 + p.16 -20
Data collection			
Interview guide	17	Were questions, prompts, guides provided by the authors? Was it pilot tested?	N/A
Repeat interviews	18	Were repeat inter views carried out? If yes, how many?	N/A
Audio/visual recording	19	Did the research use audio or visual recording to collect the data?	N/A
Field notes	20	Were field notes made during and/or after the inter view or focus group?	N/A
Duration	21	What was the duration of the inter views or focus group?	p. 5
Data saturation	22	Was data saturation discussed?	N/A
Transcripts returned	23	Were transcripts returned to participants for comment and/or	N/A

Topic	Item No.	Guide Questions/Description	Reported on Page No.
		correction?	
**Domain 3: analysis and findings**			
Data analysis			
Number of data coders	24	How many data coders coded the data?	p. 9
Description of the coding tree	25	Did authors provide a description of the coding tree?	N/A
Derivation of themes	26	Were themes identified in advance or derived from the data?	p. 9
Software	27	What software, if applicable, was used to manage the data?	p. 9
Participant checking	28	Did participants provide feedback on the findings?	N/A
Reporting			
Quotations presented	29	Were participant quotations presented to illustrate the themes/findings? Was each quotation identified? e.g. participant number	p. 16-20
Data and findings consistent	30	Was there consistency between the data presented and the findings?	p. 16-20
Clarity of major themes	31	Were major themes clearly presented in the findings?	p. 16-20
Clarity of minor themes	32	Is there a description of diverse cases or discussion of minor themes?	p. 19-20

Developed from: Tong A, Sainsbury P, Craig J. Consolidated criteria for reporting qualitative research (COREQ): a 32-item checklist for interviews and focus groups. *International Journal for Quality in Health Care*. 2007. Volume 19, Number 6: pp. 349 – 357

Once you have completed this checklist, please save a copy and upload it as part of your submission. DO NOT include this checklist as part of the main manuscript document. It must be uploaded as a separate file.

Figure S3. Diagnostics plausibility and stability of imputed values

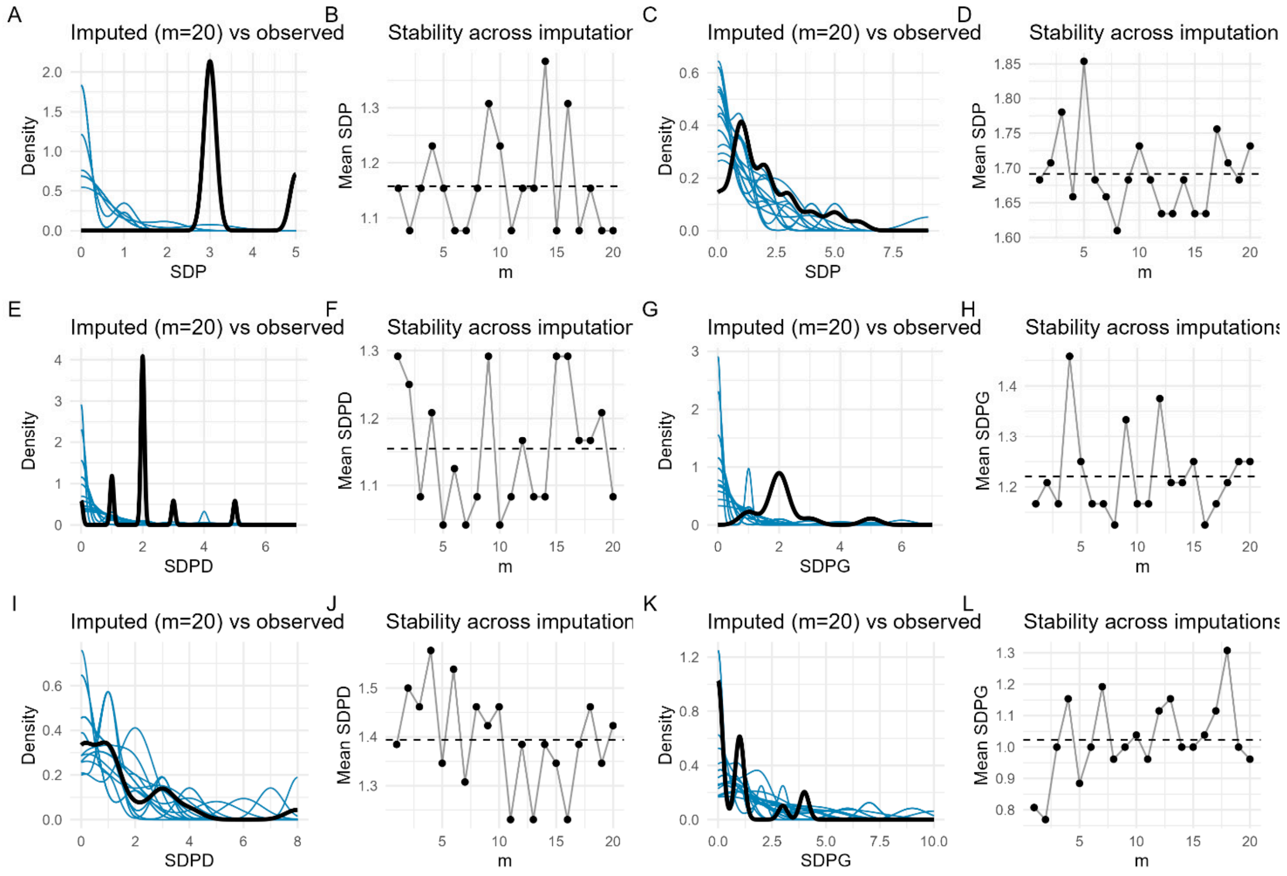

Figure S4. Sensitivity tipping point analysis under missing-at-random assumption

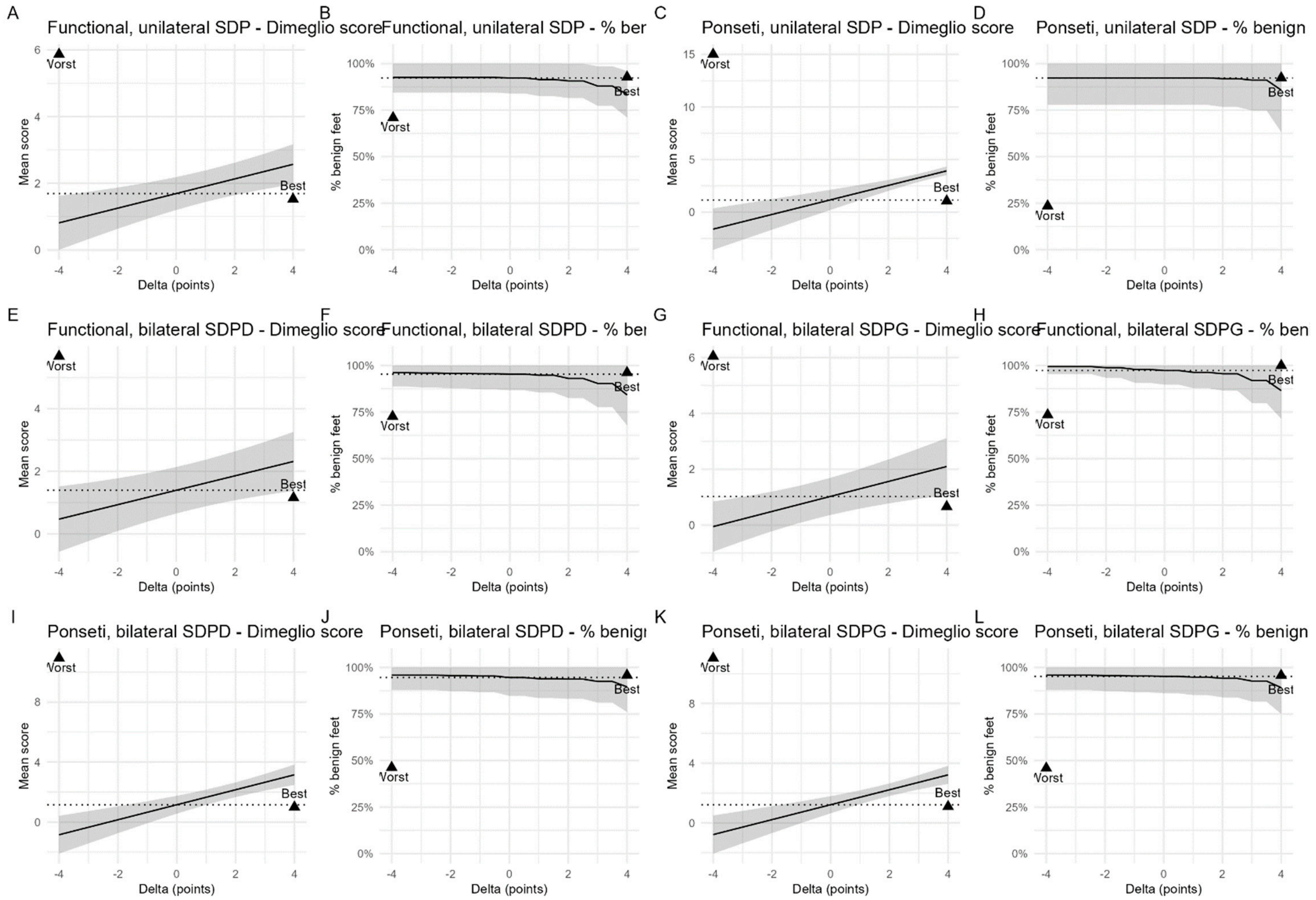

Table S1.

Unit costs and data sources for health care consumption.

Resource	Unit cost (€ 2023)	Data source for unit cost valuation	Data source for data collection
**Act**			
Rehabilitation of a limb and its root, whatever the nature and location of the pathology treated			
Unilateral	15.05/16.13		CRF
Bilateral	19.35/20.43		CRF
Manipulation of varus equinus clubfoot or convex foot, with production of a rigid external progressive reduction appliance	94.46	CCAM	CRF
Unilateral radiography of the foot with 1 to 3 views	19.95	CCAM	CRF
Lengthening of a hindfoot tendon using a direct approach	155.98	CCAM	CRF
**Material Ponseti**			
Orthopaedic correction, foot, open-toe shoe	24.04	LPP	CRF
**Material Functional**			
Denis Browne splint plates	30.00	LPP	CRF
Rigid polyolefin boot without anterior valve	479.45	LPP	CRF

Thermoformed cruro-pedic splint - Leg with foot (knee to toes).	56.96	LPP	CRF
SOFT CAST resin strip	10.50	LPP	CRF
**Inpatient stays**			
Other musculoskeletal and connective tissue diseases, level 1	1,862.35	ENC	CRF
Other pathologies of the musculoskeletal system and connective tissue, very short stay	778.19	ENC	CRF

Table S2.

Proportion of missing data (functional method).

Visit number	Unilateral clubfoot				Bilateral clubfoot			
	Variable name							
	DeMiglio score	Splint type	Out-of-pocket costs	Medical consultations	DeMiglio score – right foot	Dimeglio score – left foot	Splint type	Out-of-pocket costs
1	0.0%	0.0%	0.0%	0.0%	0.0%	0.0%	0.0%	0.0%
2	15.4%	0.0%	0.0%	7.7%	25.0%	25.0%	0.0%	0.0%
3	15.4%	0.0%	0.0%	7.7%	25.0%	25.0%	0.0%	0.0%
4	15.4%	0.0%	15.4%	7.7%	25.0%	25.0%	0.0%	4.2%
5	15.4%	7.7%	0.0%	0.0%	25.0%	25.0%	0.0%	8.3%
6	23.1%	7.7%	0.0%	23.1%	29.2%	29.2%	87.5%	16.7%
7	30.8%	7.7%	23.1%	23.1%	29.2%	29.2%	8.3%	12.5%
8	23.1%	0.0%	30.8%	23.1%	29.2%	29.2%	12.5%	41.7%
9	46.2%	0.0%	46.2%	0.0%	58.3%	58.3%	0.0%	58.3%
10	0.0%	0.0%	7.7%	15.4%	25.0%	25.0%	16.7%	41.7%
11	15.4%	7.7%	23.1%	15.4%	25.0%	25.0%	8.3%	37.5%
12	30.8%	15.4%	30.8%	0.0%	33.3%	33.3%	12.5%	37.5%
13	69.2%	0.0%	0.0%	0.0%	66.7%	66.7%	45.8%	66.7%
14	69.2%	23.1%	69.2%	0.0%	50.0%	50.0%	29.2%	50.0%

Proportion of missing data (Ponseti method).

Visit number	Unilateral clubfoot	Bilateral clubfoot		
	Variable name			
	DeMiglio score	DeMiglio score – right foot	Dimeglio score – left foot	Splint type

1	0.0%	0.0%	0.0%	0.0%
2	34.2%	30.8%	34.6%	0.0%
3	2.4%	0.0%	0.0%	0.0%
4	2.4%	0.0%	0.0%	0.0%
5	2.4%	3.9%	3.9%	3.9%
6	2.4%	15.4%	19.2%	3.9%
7	22.0%	11.5%	11.5%	11.5%
8	22.0%	23.1%	26.9%	7.7%

Table S3

Complete-case, multiply imputed, worst-case, and best-case estimates of benign-foot proportion at 24 months.

label	foot	cc_estimate	mi_estimate	worst	worst_lower	worst_upper	best	best_lower	best_upper
Functional, unilateral	SDP	0,90625	0,923170732	0,708415	0,585365854	0,853658537	0,928	0,853658537	1
Ponseti, unilateral	SDP	0,75	0,923076923	0,237846	0,076923077	0,461538462	0,924154	0,769230769	1
Functional, bilateral	SDPD	0,95	0,953846154	0,728615	0,576923077	0,884615385	0,9585	0,847130964	1
Functional, bilateral	SDPG	1	0,973076923	0,731	0,538461538	0,884615385	1		
Ponseti, bilateral	SDPD	0,916666667	0,945833333	0,456917	0,25	0,665608122	0,9565	0,875	1
Ponseti, bilateral	SDPG	0,916666667	0,952083333	0,452667	0,25	0,625	0,958667	0,875	1

Table S4

Sub-groups analysis: consequences for unilateral clubfeet, by severity at inclusion (n=53).

	**Patients benign, moderate or severe clubfoot at inclusion**			**Patients with a very severe clubfoot at inclusion**		
	Functional method (*n* = 29)	Ponseti method (*n* = 11)	Difference (Ponseti - Functional)	Functional method (*n* = 11)	Ponseti method (*n* = 2)	Difference (Ponseti - Functional)
**Mean Dimeglio score at 24 months**	1.7 (1.1; 2.4)	0 .8 (0; 1.6)	- 0.9 (- 1.9; 0.2)	1.9 (1.5; 2.4)	2.8 (0.0; 5.0)	0.9 (-2.2; 3.4)
**Percentage of patients with a benign clubfoot at 24 months**	89.0 (75.9; 100.0)	100.0 (100.0; 100.0)	11.0 (0.0; 24.1)	100.0 (100.0; 100.0)	50.0 (0.0; 100.0)	- 50.0 (- 100.0; 0.0)

Table S5

Sub-groups analysis: consequences for bilateral clubfeet, by severity at inclusion (n=47).

	**Patients benign, moderate or severe clubfoot at inclusion**			**Patients with a very severe clubfoot at inclusion**		
	Functional method (*n* = 16)	Ponseti method (*n* = 4)	Difference (Ponseti - Functional)	Functional method (*n* = 9)	Ponseti method (*n* = 20)	Difference (Ponseti - Functional)
**Left foot**						
Mean Dimeglio score at 24 months	0.3 (0.1; 0.6)	0.01 (0.0; 0.3)	-0.3 (-0.6; -0.1)	2.2 (0.9; 3.9)	1.5 (0.9; 2.1)	-0.8 (-2.5; 0.7)
Percentage of patients with a benign clubfoot at 24 months	100.0 (100.0; 100.0)	100.0 (100.0; 100.0)	0.0 (0.0; 0.0)	92.6 (66.7; 100.0)	94.2 (80.0; 100.0)	1.4 (-15.0; 28.3)
**Right foot**						
Mean Dimeglio score at 24 months	0.5 (0.2; 0.9)	0.0 (0.0; 0.0)	-0.5 (-0.9; -0.2)	3.1 (1.8; 4.7)	1.4 (0.8; 2.1)	-1.8 (-3.4; -0.3)
Percentage of patients with a benign clubfoot at 24 months	100.0 (100.0; 100.0)	100.0 (100.0; 100.0)	0.0 (0.0; 0.0)	86.8 (55.6; 100.0)	93.5 (80.0; 100.0)	-6.7 (-15.0; 34.4)
